# Nitric Oxide Functions as a Key Mediator in Brassinosteroid-Enhanced Alkaline Tolerance in Cucumber

**DOI:** 10.3390/plants14213367

**Published:** 2025-11-03

**Authors:** Wenjing Nie, Peng Qiao, Yinyu Gu, Qitong Huang, Jie Wang, Haiman Ge, Chi Zhang, Qinghua Shi

**Affiliations:** 1 Shandong Engineering Research Center of Functional Crop Germplasm Innovation and Cultivation Utilization, Yantai Engineering Research Center for Plant Stem Cell Targeted Breeding, Shandong Institute of Sericulture, Yantai 264001, China; cottage1990@163.com (W.N.);; 2 National Center of Technology Innovation for Comprehensive Utilization of Saline-Alkali Land, Dongying 257347, China; 3 Stage Key Laboratory of Crop Biology, College of Horticulture Science and Engineering, Shandong Agricultural University, Tai’an 271018, China

**Keywords:** cucumber, alleviating effect, alkaline stress, 2,4-epibrassinolide, nitric oxide

## Abstract

This study investigated how exogenous 2,4-epibrassinolide (EBR) and nitric oxide (NO) enhance the tolerance of cucumber (*Cucumis sativus* L.) seedlings to NaHCO_3_-induced alkaline stress under hydroponic conditions. NaHCO_3_ exposure caused severe sodium toxicity, reactive oxygen species (ROS) accumulation, and photosynthetic inhibition, which, together, suppressed plant growth. Treatments with either EBR or NO significantly improved plant performance by alleviating these adverse effects. Both regulators enhanced the ROS scavenging system, maintained ionic homeostasis, and alleviated sodium toxicity. They also stimulated the activities of vacuolar H^+^-ATPase, H^+^-PPase, and plasma membrane H^+^-ATPase, and increased the accumulation of citric and malic acids, thereby sustaining higher photosynthetic efficiency under stress conditions. qRT-PCR analysis further revealed that EBR and NO upregulated *SOS1* and *NHX2* (sodium transporters) as well as *PIP1;2* and *PIP2;4* (aquaporins), confirming their involvement in ionic and osmotic regulation. Pharmacological experiments showed that application of NO synthesis inhibitors, including tungstate and L-NAME, as well as the NO scavenger cPTIO, markedly weakened the protective effects of EBR. In contrast, application of the brassinosteroid biosynthesis inhibitor brassinazole (BRz) only had a limited effect on NO-mediated stress tolerance. Collectively, these findings demonstrate that NO functions as a downstream signaling mediator of EBR, coordinating multiple defense pathways including photosynthetic regulation, antioxidant protection, ion balance, aquaporin activity, and organic acid metabolism to enhance cucumber resistance to alkaline stress.

## 1. Introduction

Crops are frequently exposed to various abiotic stresses, which not only reduce yield but also cause major economic losses. Among these stresses, salt–alkali stress is regarded as one of the most detrimental factors limiting global crop productivity. This type of stress is generally divided into two categories: alkaline and neutral salt stress [1,2]. Reports indicate that salt–alkali conditions affect more than 800 million hectares of land worldwide, accounting for about 60% of arable farmland, and approximately 434 million hectares of these soils are characterized by strong alkalinity [3,4]. Plant growth is seriously affected under salt–alkali conditions because of disrupted osmotic balance, ionic toxicity, and the excessive generation of reactive oxygen species (ROS), which together lead to oxidative damage [3,4]. Under such conditions, the accumulation of sodium ions (Na^+^) in the rhizosphere often results in potassium (K^+^) deficiency and severe nutrient imbalance [5]. Alkaline stress, caused mainly by sodium carbonate (Na_2_CO_3_) and sodium bicarbonate (NaHCO_3_), is usually more harmful than neutral salt stress, which is mainly associated with sodium chloride (NaCl) and sodium sulfate (Na_2_SO_4_). This difference in severity is largely due to the high pH of alkaline stress, which causes the precipitation of essential ions such as magnesium (Mg^2+^) and phosphate (H_2_PO_4_^−^), further aggravating ionic disequilibrium, metabolic disturbance, and oxidative injury [6,7]. In recent years, researchers have paid increasing attention to how plants adapt to alkaline environments, especially in relation to signal transduction and redox regulation mechanisms [4,8,9]. However, the physiological and molecular bases of plant tolerance to alkaline stress remain unclear, and more detailed studies are still needed to explain how plants coordinate these adaptive responses.

Nitric oxide (NO), a volatile signaling molecule with high diffusivity and nonpolar characteristics, is an important endogenous regulator in plants [10]. It participates in a wide range of physiological and biochemical processes, including seed germination, flowering, ion homeostasis, and stress signaling [11]. NO also functions as a key messenger in redox regulation by promoting the removal of reactive oxygen species (ROS) [6]. Exogenous application of NO has been shown to improve plant stress tolerance by enhancing antioxidant capacity and activating defense responses under adverse environmental conditions [12].

Brassinosteroids (BRs) are naturally occurring plant steroid hormones containing multiple hydroxyl groups. They play essential roles in plant growth and development, regulating photomorphogenesis, cell elongation, and hormone synthesis [13,14]. Beyond developmental regulation, BRs markedly improve plant tolerance to a variety of environmental stresses, including drought, salinity, extreme temperatures, and heavy metal toxicity, as well as biotic stresses such as pathogen attack [13,14]. Previous studies have demonstrated that BRs modulate gene expression by influencing both structural genes and transcriptional regulators, thereby enhancing the plant’s capacity to adapt to stress [14]. BRs also activate antioxidant defense systems by stimulating enzymes such as superoxide dismutase (SOD), catalase (CAT), and ascorbate peroxidase (APX), which are essential for maintaining cellular redox homeostasis during stress [15,16]. Furthermore, studies have elucidated the BR signaling cascade, which begins at the membrane receptor level and proceeds through cytoplasmic kinases to nuclear transcription factors [17,18,19]. Through these regulatory networks, BRs promote balanced metabolism and improve crop productivity by reinforcing stress adaptation and optimizing growth-related processes [15,16,19].

Cucumber (*Cucumis sativus* L.) is an agronomically important vegetable crop cultivated worldwide, yet it is highly sensitive to sodic alkaline stress [20,21]. Previous studies from our group demonstrated that exogenous application of either 24-epibrassinolide (EBR, a highly active BR analog) or NO enhances plant tolerance to alkaline stress [22,23]. However, the potential interaction between BRs and NO in regulating cucumber responses to alkaline stress has not been fully elucidated. In this study, we investigated how EBR and NO interact to modulate cucumber seedling responses under NaHCO_3_-induced alkaline conditions. Our results showed that the stress-alleviating effect of EBR was markedly reduced when NO generation was inhibited by specific scavengers or biosynthesis inhibitors, whereas the protective effect of NO application remained unchanged when BR biosynthesis was disrupted. These observations strongly suggest that NO functions downstream of BR signaling. Overall, this study provides evidence that NO acts as a downstream mediator in BR-regulated tolerance to alkaline stress, thereby enhancing antioxidant defense, maintaining ion balance, improving osmotic stability, and contributing to rhizospheric pH regulation.

## 2. Results

### 2.1. Exogenous EBR and NO Enhanced Cucumber Tolerance to NaHCO_3_ Stress

**Figure 1:**
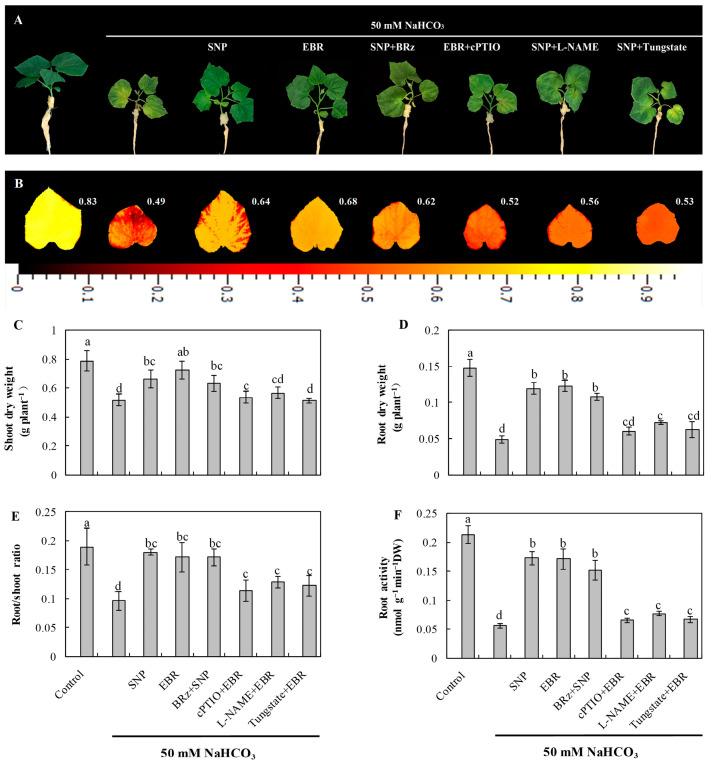
Effects of EBR and NO on the growth of cucumber seedlings under NaHCO_3_ stress. (**A**) Phenotypic appearance of seedlings after 8 days of exposure; (**B**) chlorophyll fluorescence images showing the maximum quantum efficiency of photosystem II (Fv/Fm); (**C**) shoot dry weight; (**D**) root dry weight; (**E**) root-to-shoot ratio; (**F**) root activity. Different lowercase letters indicate statistically significant differences among treatments at the 0.05 level (*p* < 0.05, n = 3). Note that changes to the position of figures and tables may occur during the final steps.

As shown in Figure 1A, NaHCO_3_ stress caused pronounced chlorosis and wilting of cucumber seedling leaves and significantly reduced the accumulation of shoot and root dry weight (Figure 1C,D). In addition, NaHCO_3_ stress decreased the maximum quantum efficiency of photosystem II (Fv/Fm) (Figure 1B), the root-to-shoot ratio (Figure 1E), and root activity (Figure 1F). Exogenous application of EBR or the NO donor sodium nitroprusside (SNP) effectively alleviated the damage induced by NaHCO_3_ stress. Both treatments enhanced shoot and root dry weight accumulation, root-to-shoot ratio, and root activity under stress conditions. Furthermore, the protective effect of SNP was significantly inhibited by the NO scavenger 2-(4-carboxyphenyl)-4,4,5,5-tetramethylimidazoline-1-oxyl-3-oxide (cPTIO), the nitrate reductase (NR) inhibitor tungstate [Na_2_WO_4_·2H_2_O], and the NO synthase (NOS) inhibitor Nω-nitro-L-arginine methyl ester (L-NAME), whereas the BR biosynthesis inhibitor brassinazole (BRz) had little effect on the protective role of SNP. These results indicate that NO may act as a downstream signaling molecule in BR-mediated regulation of alkaline tolerance in cucumber seedlings, thereby mitigating the inhibitory effects of alkaline stress on plant growth.

### 2.2. EBR and NO Maintain Photosynthetic Efficiency Under NaHCO_3_ Stress

As shown in Table 1, NaHCO_3_ stress caused a pronounced decline in Pn and pigment contents (Chl a, Chl b, and Car), accompanied by a reduction in Fv/Fm (Figure 1B), reflecting the inhibition of photosystem II (PSII) photochemical activity. In contrast, EBR and NO treatments significantly improved photosynthetic parameters under NaHCO_3_ exposure, indicating a restoration of PSII efficiency and chlorophyll stability. Moreover, the BR-induced increase in photosynthetic efficiency was strongly suppressed by cPTIO, tungstate, and L-NAME, confirming the involvement of NO in BR-mediated photoprotection. Conversely, BRz had little effect on the NO-induced improvement in photosynthesis, reinforcing the hypothesis that NO acts as a downstream signaling molecule in BR-mediated regulation under alkaline stress.

### 2.3. EBR and NO Reduce ROS Accumulation and Lipid Peroxidation Under NaHCO_3_ Stress

**Figure 2:**
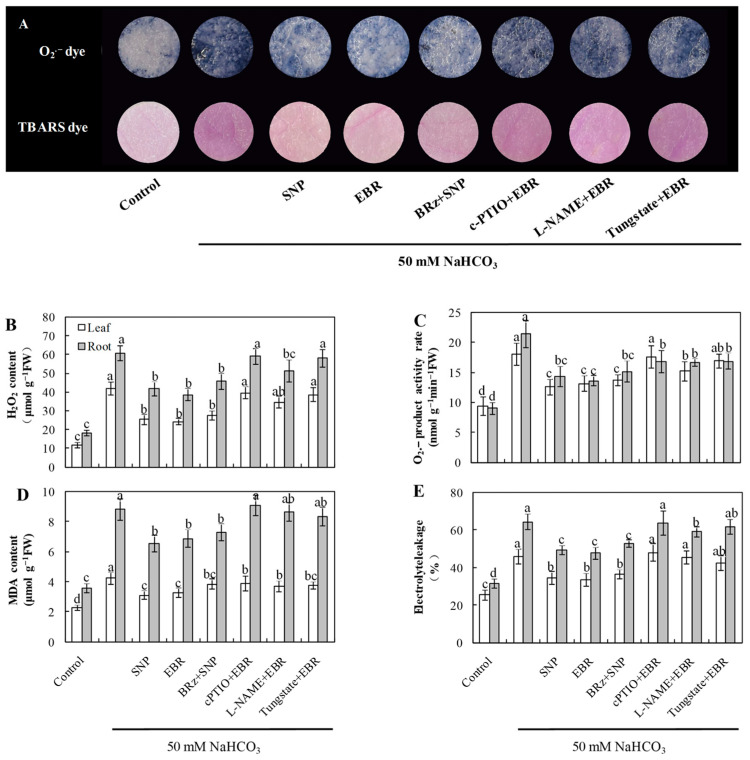
Effects of exogenous EBR and NO on oxidative stress in cucumber seedlings under NaHCO_3_ stress. (**A**) Histochemical staining showing superoxide anion (O_2_^−^) by NBT and lipid peroxidation by TBARS in cucumber leaves; (**B**) hydrogen peroxide (H_2_O_2_) content; (**C**) superoxide anion (O_2_^−^) production rate; (**D**) malondialdehyde (MDA) content; (**E**) electrolyte leakage (EL). Different lowercase letters indicate statistically significant differences among treatments at the 0.05 level (*p* < 0.05, n = 3).

As shown in Figure 2, NaHCO_3_ exposure led to a marked rise in the levels of O_2_^−^, H_2_O_2_, and MDA, accompanied by increased electrolyte leakage in both roots and leaves. The addition of EBR and NO markedly reduced ROS and MDA accumulation and decreased EL compared with NaHCO_3_ treatment alone. Notably, the EBR-induced reductions in O_2_^−^ and H_2_O_2_ levels were largely reversed or markedly weakened by cPTIO, L-NAME, or tungstate, whereas BRz treatment had minimal influence on the NO-mediated decreases in O_2_^−^ and H_2_O_2_ concentrations under NaHCO_3_ stress. Histochemical staining of ROS and TBARS in cucumber leaves further corroborated these biochemical results (Figure 2A). These observations support the possibility that NO acts as a downstream component in BR-dependent regulation of ROS detoxification in cucumber seedlings under alkaline stress.

### 2.4. EBR and NO Increase the Activities of Antioxidant Enzymes Under NaHCO_3_ Stress

**Figure 3:**
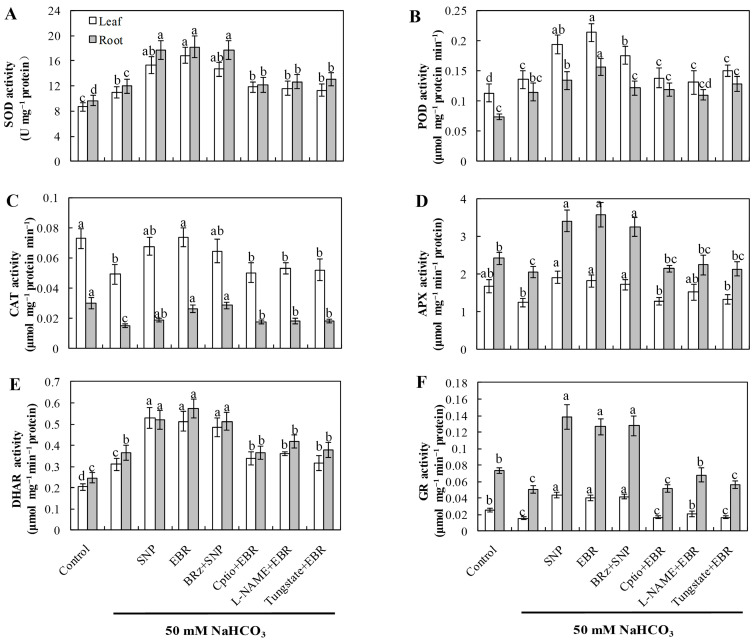
Effects of exogenous EBR and NO on antioxidant enzyme activities in cucumber seedlings under NaHCO_3_ stress. (**A**) Superoxide dismutase (SOD); (**B**) Peroxidase (POD); (**C**) Catalase (CAT); (**D**) Ascorbate peroxidase (APX); (**E**) Dehydroascorbate reductase (DHAR); (**F**) Glutathione reductase (GR). Different lowercase letters indicate statistically significant differences among treatments at the 0.05 level (*p* < 0.05, n = 3).

Compared with the control, NaHCO_3_ stress markedly enhanced the activities of SOD, POD, and DHAR, whereas the activities of CAT, APX, and GR significantly declined (Figure 3). This pattern indicates that although the antioxidant system was activated as a compensatory response, the imbalance among individual enzymes impaired the overall redox equilibrium in cucumber seedlings. Treatment with exogenous EBR or NO notably elevated the activities of all six enzymes, thereby enhancing antioxidant defense capacity and mitigating oxidative damage induced by NaHCO_3_ stress. Furthermore, the EBR-induced enhancement of antioxidant enzyme activities was markedly suppressed by cPTIO, L-NAME, and tungstate, confirming that NO synthesis is required for the BR-mediated antioxidant response. In contrast, the NO-induced enzyme activation was not significantly affected by BRz treatment. These findings collectively demonstrate that EBR enhances the antioxidant enzyme system primarily through an NO-dependent signaling cascade, contributing to improved oxidative stress tolerance under alkaline conditions.

### 2.5. EBR and NO Improve the AsA–GSH Cycle Under NaHCO_3_ Stress

**Figure 4:**
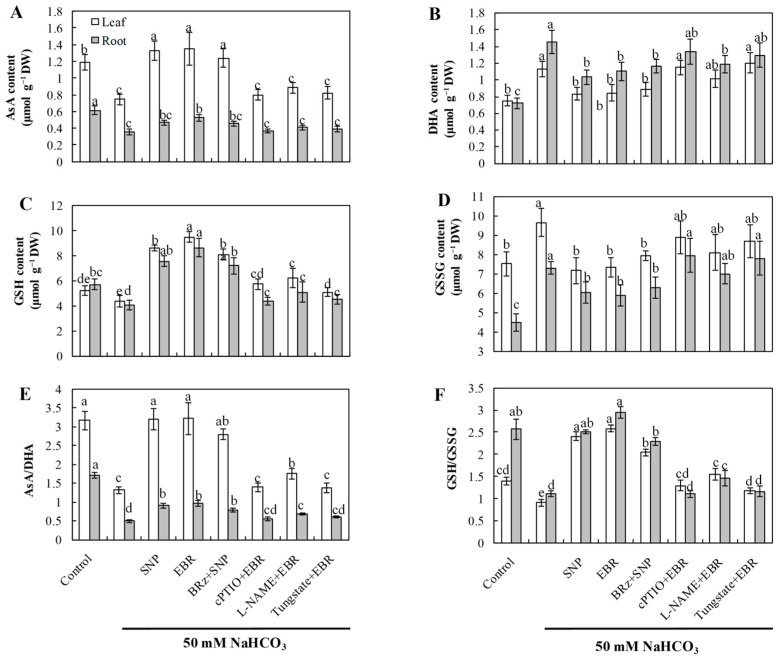
Effects of exogenous EBR and NO on the contents of non-enzymatic antioxidants in cucumber seedlings under NaHCO_3_ stress. (**A**) Ascorbic acid (AsA); (**B**) Dehydroascorbic acid (DHA); (**C**) Reduced glutathione (GSH); (**D**) Oxidized glutathione (GSSG); (**E**) AsA/DHA ratio; (**F**) GSH/GSSG ratio. Different lowercase letters indicate statistically significant differences among treatments at the 0.05 level (*p* < 0.05, n = 3).

NaHCO_3_ stress markedly decreased the concentrations of AsA and GSH, along with a significant reduction in the AsA/DHA and GSH/GSSG ratios, indicating a disrupted redox state in the AsA–GSH cycle (Figure 4). Exogenous application of EBR and NO substantially increased AsA and GSH levels and improved both AsA/DHA and GSH/GSSG ratios, thereby restoring cellular redox homeostasis and enhancing antioxidant buffering capacity under alkaline conditions. Importantly, the beneficial effects induced by EBR were largely abolished by treatments with cPTIO, L-NAME, and tungstate, while BRz had minimal effect on the NO-mediated improvements. These results suggest that EBR enhances the AsA–GSH cycle predominantly through NO-dependent signaling, thereby maintaining redox equilibrium during NaHCO_3_ stress.

### 2.6. EBR and NO Improve Ion Homeostasis Under NaHCO_3_ Stress

**Figure 5:**
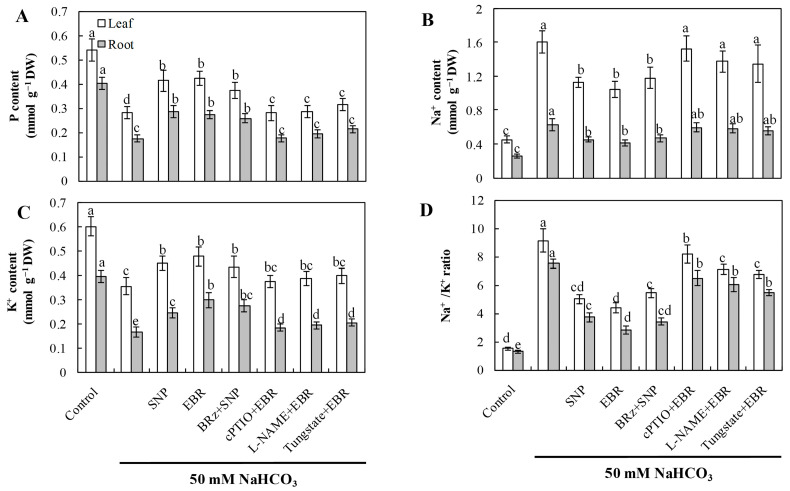
Effects of exogenous EBR and NO on ion homeostasis in cucumber seedlings exposed to NaHCO_3_ stress. (**A**) Phosphorus (P); (**B**) sodium (Na^+^); (**C**) potassium (K^+^); (**D**) Na^+^/K^+^ ratio in leaves and roots. Different lowercase letters indicate statistically significant differences among treatments at the 0.05 level (*p* < 0.05, n = 3).

NaHCO_3_ stress caused substantial Na^+^ accumulation and significantly reduced K^+^ and P contents, resulting in a markedly elevated Na^+^/K^+^ ratio (Figure 5). Exogenous application of EBR and NO effectively reduced Na^+^ accumulation, promoted K^+^ uptake, and increased P levels, thus maintaining ion homeostasis in both leaves and roots. The EBR-induced restoration of ion homeostasis was strongly inhibited by cPTIO, L-NAME, and tungstate, whereas BRz exhibited little effect on the NO-mediated regulation. These observations indicate that NO functions as a downstream signaling molecule of EBR to regulate Na^+^ and K^+^ transport, contributing to improved ion homeostasis under alkaline stress.

### 2.7. EBR and NO Upregulate Na^+^ Detoxification-Related Genes Under NaHCO_3_ Stress

**Figure 6:**
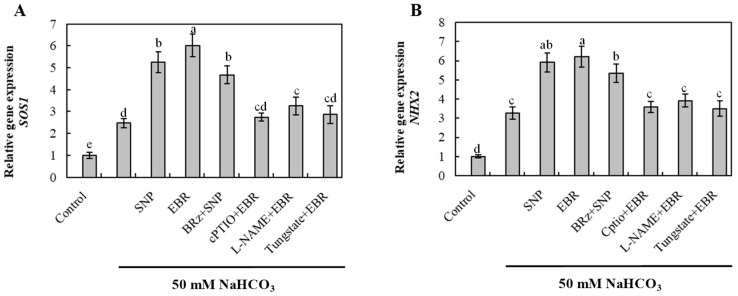
Effects of exogenous EBR and NO on the expression of Na^+^ detoxification-related genes in cucumber roots under NaHCO_3_ stress. (**A**) Salt overly sensitive 1 (*SOS1*); (**B**) Na^+^/H^+^ exchanger 2 (*NHX2*). Different lowercase letters indicate statistically significant differences among treatments at the 0.05 level (*p* < 0.05, n = 3).

To elucidate the molecular mechanism involved in Na^+^ detoxification, the transcript levels of *SOS1* (plasma membrane Na^+^/H^+^ antiporter) and *NHX2* (vacuolar Na^+^/H^+^ exchanger) were quantified (Figure 6). NaHCO_3_ stress significantly upregulated the expression of both genes, suggesting activation of Na^+^ extrusion and vacuolar compartmentalization processes. EBR and NO treatments further enhanced *SOS1* and *NHX2* expression, whereas the EBR-induced upregulation was strongly inhibited by cPTIO, L-NAME, and tungstate. Conversely, BRz had a negligible effect on the NO-mediated response. These results indicate that EBR activates Na^+^ detoxification-related genes primarily through an NO-dependent signaling pathway, thereby effectively strengthening Na^+^ efflux and vacuolar sequestration in cucumber roots.

### 2.8. EBR and NO Enhance H^+^-Pump Activities Under NaHCO_3_ Stress

**Figure 7:**
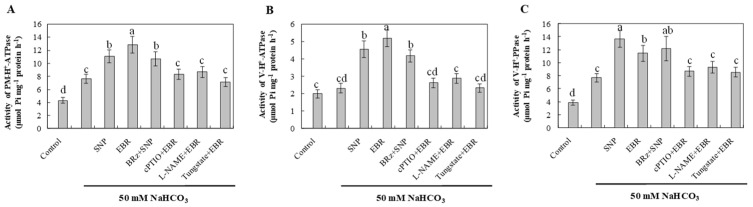
Effects of exogenous EBR and NO on proton-pump activities in cucumber roots under NaHCO_3_ stress. (**A**) Plasma membrane H^+^-ATPase (PM H^+^-ATPase) activity; (**B**) Tonoplast (vacuolar) H^+^-ATPase (V-ATPase) activity; (**C**) Tonoplast H^+^-pyrophosphatase (H^+^-PPase) activity. Different lowercase letters indicate statistically significant differences among treatments at the 0.05 level (*p* < 0.05, n = 3).

NaHCO_3_ stress increased the activities of plasma membrane H^+^-ATPase, tonoplast H^+^-ATPase, and H^+^-PPase compared with the control (Figure 7). This enhancement likely reflects an adaptive response that facilitates Na^+^ extrusion and vacuolar sequestration, helping to maintain ionic balance under alkaline conditions. Treatment with EBR or NO further stimulated the activities of these proton pumps, suggesting that both regulators enhance proton transport and ion compartmentalization in response to NaHCO_3_ stress. The activation of these enzymes by EBR was markedly suppressed by cPTIO, L-NAME, and tungstate, whereas BRz had a relatively small effect on the NO-mediated enhancement. These results indicate that NO functions as a downstream signaling molecule of EBR. The enhanced proton pump activities induced by either EBR or NO contribute to Na^+^ efflux, vacuolar compartmentalization, and overall ion homeostasis, thereby improving the alkaline tolerance of cucumber roots.

### 2.9. EBR and NO Promote Organic Acid Accumulation Under NaHCO_3_ Stress

**Figure 8:**
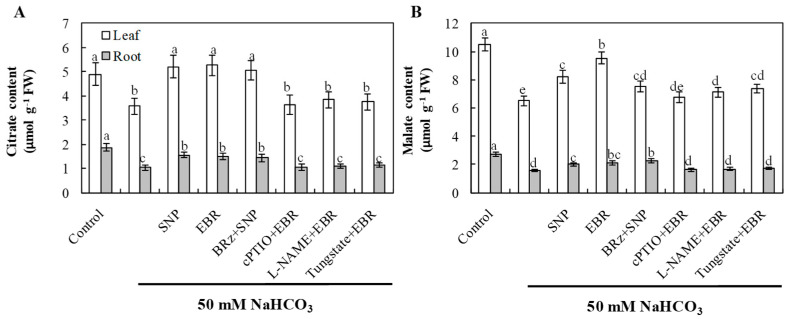
Effects of exogenous EBR and NO on organic acid accumulation in cucumber seedlings under NaHCO_3_ stress. (**A**) Citrate; (**B**) malate in roots and leaves. Different lowercase letters indicate statistically significant differences among treatments at the 0.05 level (*p* < 0.05, n = 3).

The elevated contents of citrate and malate may play important roles in buffering cytoplasmic pH and chelating excess Na^+^, thereby alleviating high-pH stress and maintaining ion homeostasis. NaHCO_3_ stress significantly decreased the levels of citrate and malate in both leaves and roots, indicating inhibition of organic acid metabolism under alkaline conditions (Figure 8). Application of EBR or NO markedly increased the accumulation of these organic acids compared with NaHCO_3_ treatment alone. The EBR-induced enhancement of organic acid accumulation was markedly suppressed by cPTIO, L-NAME, and tungstate, whereas BRz had only a slight effect on the NO-induced increases in citrate. Although BRz also reduced the NO-induced accumulation of malate, the extent of reduction was relatively small. These findings suggest that exogenous BR regulates organic acid accumulation under alkaline stress through both NR- and NOS-dependent pathways, but NO does not act entirely as a downstream signal of BR in this regulatory process.

### 2.10. EBR and NO Enhance Aquaporin Gene Expression Under NaHCO_3_ Stress

**Figure 9:**
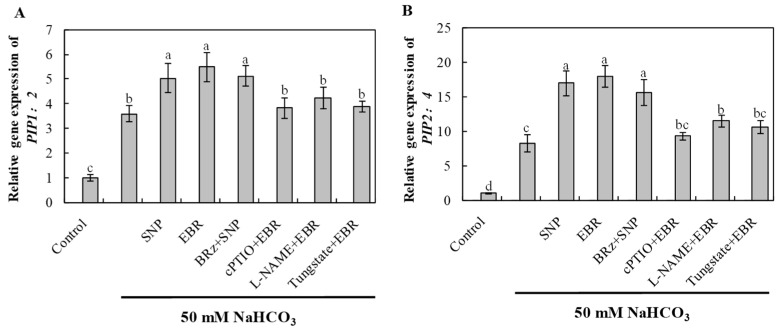
Effects of exogenous EBR and NO on aquaporin gene expression in cucumber roots under NaHCO_3_ stress. (**A**) *PIP1;2*; (**B**) *PIP2;4* after 16 h of treatment. Different lowercase letters indicate statistically significant differences among treatments at the 0.05 level (*p* < 0.05, n = 3).

NaHCO_3_ stress significantly induced the expression of aquaporin genes *PIP1;2* and *PIP2;4*, indicating an adaptive response to maintain water balance under alkaline stress (Figure 9). EBR and NO treatments further enhanced the transcript levels of these genes, thereby facilitating water transport and root hydraulic conductivity. The EBR-induced upregulation was notably inhibited by cPTIO, L-NAME, and tungstate, whereas BRz treatment had little effect on the NO-mediated increase. These results demonstrate that BR regulates aquaporin-mediated water transport via an NO-dependent pathway, improving plant water status under NaHCO_3_ stress.

## 3. Discussion

Although NO and BRs are well known to enhance plant tolerance to abiotic stresses [22,24], their interaction under alkaline conditions remains unclear. In this study, NaHCO_3_ stress caused Na^+^ toxicity, impaired photosynthesis, and excessive ROS accumulation, leading to growth inhibition in cucumber seedlings. The application of exogenous NO or EBR effectively alleviated these negative effects by sustaining photosynthetic efficiency, maintaining ion homeostasis, enhancing antioxidant defense, and promoting the expression of aquaporin genes. The protective effect of EBR was markedly suppressed when NO biosynthesis or activity was inhibited by cPTIO, tungstate, or L-NAME, whereas BRz had little influence on NO-mediated protection, indicating that NO acts downstream of BR signaling in regulating alkaline tolerance. These findings extend previous reports on BR and NO cooperation under abiotic stress and reveal a coordinated BR–NO signaling pathway that confers tolerance to NaHCO_3_-induced alkaline stress.

Under saline–alkali stress, Na^+^ concentrations in the rhizosphere are markedly elevated. Maintaining a low Na^+^ concentration in the cytosol is essential for sustaining normal metabolic processes such as photosynthesis and ROS scavenging [25,26]. Due to their similar hydration and atomic radii, Na^+^ competitively inhibits K^+^ uptake through cation channels [27], resulting in Na^+^ accumulation, K^+^ deficiency, and a substantially increased Na^+^/K^+^ ratio in cucumber plants (Figure 5). Because plants lack a Na^+^-ATPase, Na^+^ efflux occurs exclusively via the salt overly sensitive (SOS) signaling cascade, which is crucial for plant adaptation to salt stress [28]. The SOS network consists of SOS1, SOS2, and SOS3, together with NHX transporters [29]. SOS1, localized at the plasma membrane, acts as a Na^+^/H^+^ exchanger that senses Na^+^ signals and exports excess cytosolic Na^+^ [30]. Salt stress strongly induces *SOS1* expression; overexpression enhances salt tolerance, whereas knockout mutants display hypersensitivity [31]. In parallel, NHX proteins on the vacuolar membrane mediate Na^+^ compartmentalization into vacuoles [32]. Specifically, *NHX* reduces cytosolic Na^+^ accumulation by sequestering Na^+^ into the vacuole [33,34]. NHX proteins also contribute to pH homeostasis, as *Arabidopsis nhx* mutants exhibit elevated vacuolar pH compared with wild type [35]. Therefore, Na^+^/H^+^ counter-transport plays an essential role in maintaining Na^+^ efflux and vacuolar sequestration during salt and alkaline stress [36]. Because Na^+^/H^+^ exchange is an energy-dependent process, it relies on the activity of proton pumps, including plasma membrane H^+^-ATPase, vacuolar H^+^-ATPase, and vacuolar H^+^-PPase [37].

Previous studies have reported that both NO and BR can activate these proton pumps, thereby supplying the energy required for SOS1- and NHX-mediated Na^+^ transport under saline stress [24,37,38,39,40]. Consistent with these findings, our results (Figure 7) showed that BR and NO treatments markedly increased the activities of PM H^+^-ATPase, V-ATPase, and H^+^-PPase in cucumber roots under NaHCO_3_ stress. This enhancement promoted Na^+^ extrusion via *SOS1* and vacuolar sequestration through *NHX2*, stabilizing the Na^+^/K^+^ ratio and improving ion homeostasis. Furthermore, the BR-induced stimulation of proton pumps was strongly suppressed by cPTIO, L-NAME, and tungstate, whereas BRz had only a minor influence on NO-mediated responses. These results, together with the upregulated expression of *SOS1* and *NHX2* (Figure 6), indicate that NO functions downstream of BR signaling to regulate the SOS pathway and Na^+^ detoxification. However, our findings extend this regulatory mechanism to NaHCO_3_-induced alkaline stress, suggesting that the BR–NO interaction contributes to maintaining ion balance and enhancing tolerance in cucumber seedlings.

Declines in photosynthetic pigment content and suppression of the Pn under alkaline stress largely explain the restriction of plant growth and biomass accumulation. Alkaline–salinity stress disrupts chloroplast ultrastructure, accelerates chlorophyll degradation, and inhibits carbon assimilation, resulting in decreased photosynthetic capacity and impaired growth [9,23,41]. In addition, stress-induced inhibition of respiration and photosynthesis leads to electron leakage, ROS overproduction, and lipid peroxidation, ultimately damaging the photosynthetic apparatus. Similar chloroplast disorganization and oxidative injury have been reported in *Solanum lycopersicum* and *Apocynum venetum* L. (Apocynaceae) subjected to alkaline stress [4,41], supporting the view that oxidative impairment of photosynthetic structures is a primary cause of growth inhibition. It is well established that both NO and EBR play critical roles in regulating plant adaptation to abiotic stress. The beneficial influence of these compounds on cucumber biomass during NaHCO_3_ exposure can be attributed to their ability to maintain photosynthetic efficiency. Previous studies have demonstrated that exogenous NO mitigates chlorosis under alkaline stress [4,10,34], while BRs enhance photosynthetic performance under salt stress [24]. Consistent with these findings, our results showed that NaHCO_3_ stress significantly reduced chlorophyll content and Pn (Table 2), leading to decreased biomass accumulation (Figure 1), whereas exogenous BR and NO alleviated these effects and improved leaf greenness and photosynthetic rates.

Chlorophyll fluorescence is a sensitive indicator of PSII function and has been widely used to assess chloroplast integrity and stability [42,43]. The decline in Fv/Fm values observed under NaHCO_3_ stress (Figure 1B) indicates photoinhibition and reduced PSII efficiency, likely resulting from restricted electron transport between primary (Q_a_) and secondary (Q_β_) quinone acceptors [34,44]. In contrast, exogenous BR and NO significantly increased Fv/Fm, suggesting protection of PSII reaction centers. Similar improvements in photochemical efficiency by NO or BR treatment have been observed in *Solanum lycopersicum*, *Cyclocarya paliurus*, *Abelmoschus esculentus* L. and Maize under salt and alkaline stress [34,38,45,46]. Moreover, the BR-induced enhancement in Fv/Fm was abolished by cPTIO and markedly reduced by L-NAME and tungstate, while BRz had little effect on NO-mediated protection. These findings indicate that NO acts downstream of BR signaling to preserve photosynthetic machinery under alkaline stress.

Salt and alkaline stress also impose osmotic limitations that disturb water balance in cucumber plants. Such effects arise from reduced extracellular water potential, which limits water uptake, and from impaired aquaporin activity, which restricts transmembrane water transport [47,48]. Aquaporins are key regulators of water permeability and osmotic adjustment, and their activity accounts for 70–90% of root hydraulic conductance [49]. In this study, exogenous BR and NO upregulated the transcription of aquaporin genes (Figure 9), particularly *PIP1;2* and *PIP2;4*, thereby improving root water absorption and translocation under NaHCO_3_ stress. These genes were significantly induced 16 h after treatment, and their expression was further enhanced by BR or NO application. However, the BR-induced increase was strongly suppressed by cPTIO, tungstate, and L-NAME, whereas BRz had minimal influence on NO-mediated induction. Collectively, these findings suggest that BR modulates aquaporin expression through an NO-dependent pathway, facilitating water transport and contributing to improved stress tolerance.

Salinity stress disrupts photosynthesis and respiration, leading to impaired electron transport and excessive accumulation of reactive oxygen species (ROS) [50]. At moderate levels, ROS act as signaling molecules that trigger plant defense pathways [51]; however, when produced excessively, they oxidize lipids, proteins, and nucleic acids, resulting in structural and functional damage. Thus, oxidative stress represents one of the most prominent manifestations of plant injury under saline–alkali conditions [52,53]. Previous studies have demonstrated that EBR can upregulate antioxidant-related genes and enhance enzymatic activity, thereby improving stress resistance [54,55]. In this study, NaHCO_3_ stress caused a sharp increase in ROS levels, elevated MDA content, and led to pronounced lipid peroxidation of plasma membranes (Figure 2). Exogenous EBR and NO treatments significantly reduced ROS accumulation and MDA levels, thereby alleviating membrane injury. In parallel, the activities of major antioxidant enzymes, including SOD, POD, and CAT, were enhanced in both roots and leaves during stress exposure. Enzymes related to the AsA–GSH cycle, such as APX, GR, and DHAR, also showed increased activities (Figure 3), accompanied by higher AsA and GSH contents (Figure 4). As a result, the NaHCO_3_-induced accumulation of DHA and GSSG was mitigated, leading to improved AsA/DHA and GSH/GSSG ratios and a more balanced redox state. These changes maintained efficient operation of the AsA–GSH cycle and enhanced the overall ROS-scavenging capacity of cucumber seedlings.

The responses observed align with previous reports showing that EBR enhances antioxidant metabolism under abiotic stress [22,53]. In our study, the EBR-induced activation of the antioxidant system was clearly dependent on endogenous NO. When NO biosynthesis or signaling was blocked by cPTIO, tungstate, or L-NAME, the protective effects of EBR on enzyme activities and non-enzymatic antioxidants were markedly weakened. In contrast, the BR biosynthesis inhibitor BRz had little influence on the NO-induced antioxidant response (Figure 2, Figure 3 and Figure 4), indicating that NO acts downstream of EBR rather than as a parallel regulator. Previous research has shown that NO can modulate antioxidant enzymes [4,19,23], while BRs promote ROS detoxification by stimulating NO generation and enhancing the AsA–GSH recycling system [38,54]. Together with our results, these findings suggest that EBR enhances antioxidant capacity through an NO-dependent signaling pathway, which helps to mitigate oxidative damage and maintain redox homeostasis under NaHCO_3_ stress.

The accumulation of organic acids represents an important adaptive strategy for plants under adverse environmental conditions. In saline–alkali environments, organic acids act as intracellular pH buffers that counteract the effects of high alkalinity, thereby protecting metabolic activity and maintaining ion balance [56,57]. Among these, citric acid and malic acid are the predominant organic acids in cucurbit crops, functioning both as pH regulators and as intermediates in the tricarboxylic acid (TCA) cycle [58,59]. In our study, NaHCO_3_ stress led to a marked decline in the contents of citrate and malate (Figure 8), while the application of exogenous EBR or NO significantly increased their accumulation, alleviating high pH–induced damage. Enhanced organic acid biosynthesis under these treatments likely helped to stabilize cellular pH and facilitate ion homeostasis. Similar results have been reported in cotton and rice, where exogenous hormones or signaling molecules promoted organic acid metabolism to enhance tolerance to alkaline stress [60,61]. The promotive effect of EBR on citrate and malate accumulation was largely inhibited by cPTIO, tungstate, and L-NAME, whereas BRz had little influence on the NO-induced response (Figure 8). These results indicate that the regulation of organic acid metabolism by EBR is mediated through NO-dependent signaling rather than direct stimulation. This conclusion is consistent with the observed downstream role of NO in EBR-induced ion and redox regulation. Together, these findings suggest that EBR enhances alkaline tolerance in cucumber by activating NO signaling to stimulate organic acid synthesis and pH-buffering capacity, thereby maintaining metabolic stability under NaHCO_3_ stress.

**Figure 10:**
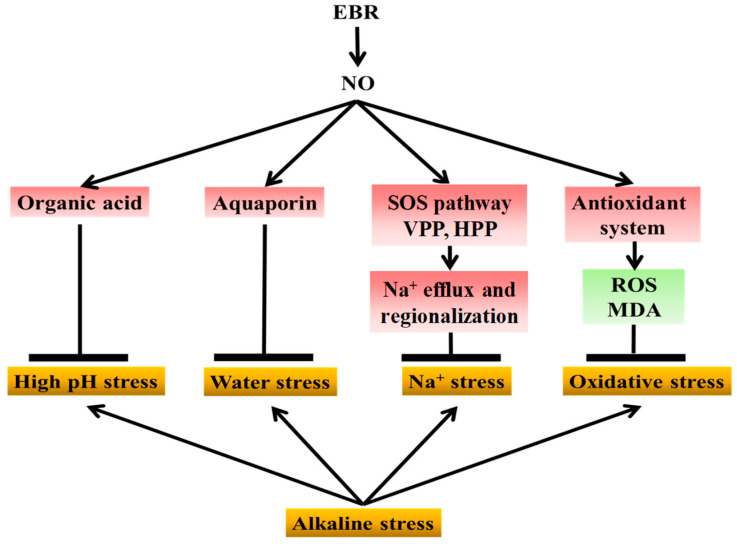
Proposed model of NO-mediated EBR regulation of cucumber tolerance to alkaline stress. NaHCO_3_ stress induces sodium toxicity, oxidative imbalance, high-pH stress, and water stress, thereby inhibiting cucumber growth. Exogenous EBR enhances stress tolerance by relying on NO as a downstream signaling molecule to coordinate multiple defense pathways. EBR-induced NO signaling promotes organic acid accumulation to buffer cellular pH and alleviate high-pH stress; enhances aquaporin expression to relieve water stress; activates the SOS pathway together with H^+^-ATPase and H^+^-PPase activities to facilitate sodium efflux and vacuolar compartmentalization; and stimulates the antioxidant system, including antioxidant enzymes and the ascorbate–glutathione cycle, to reduce reactive oxygen species accumulation and oxidative damage. These coordinated effects collectively mitigate growth inhibition in cucumber seedlings exposed to sodium bicarbonate stress (The yellow boxes represent the types of physiological damage caused by alkaline stress; the red boxes indicate factors upregulated by exogenous NO and EBR; and the green boxes indicate factors downregulated by NO and EBR).

Collectively, and as illustrated in Figure 10, these findings demonstrate that exogenous EBR enhances the tolerance of cucumber seedlings to NaHCO_3_-induced alkaline stress through NO-dependent signaling. NaHCO_3_ stress causes Na^+^ toxicity, oxidative imbalance, water stress, and high-pH-induced metabolic inhibition. As a downstream signal, NO transmits EBR regulation to multiple defense pathways, including antioxidant protection, ion homeostasis, aquaporin activity, and organic acid metabolism, thereby alleviating oxidative damage, ionic imbalance, water stress, and high-pH injury. This coordination maintains cellular stability and higher photosynthetic efficiency, ultimately supporting plant growth under alkaline conditions. Overall, NO acts as a central mediator linking BR perception to physiological and metabolic adjustments that collectively reduce alkaline-induced injury in cucumber. This work elucidates the signaling pathway by which EBR mitigates saline-alkali stress and further clarifies the mechanistic relationship between EBR and NO under abiotic stress.

## 4. Materials and Methods

### 4.1. Plant Growth Conditions and Treatment Setup

Seeds of cucumber (*Cucumis sativus* L. cv. Jinyan 4) were first placed on moist filter paper at 28 °C for 24 h to promote germination. The sprouted seeds were then transferred into vermiculite within a growth chamber. Seedlings were maintained under controlled conditions (25 °C/18 °C day/night) with a 14 h light/10 h dark photoperiod for 8 days. The NaHCO_3_ concentration (50 mM) was determined based on preliminary tests. Once the first true leaf had fully expanded, uniform seedlings were selected and individually transplanted into 0.8 L plastic pots containing Hoagland nutrient solution (one seedling per pot). Following an 8-day acclimatization period, experimental treatments were initiated.

The study included eight treatment groups:

- Control—Hoagland nutrient solution only.
- NaHCO_3_—Hoagland solution supplemented with 50 mM NaHCO_3_.
- NaHCO_3_ + EBR—50 mM NaHCO_3_ combined with 0.2 μM 24-epibrassinolide (EBR).
- NaHCO_3_ + SNP—50 mM NaHCO_3_ plus 100 μM sodium nitroprusside (SNP, an NO donor).
- NaHCO_3_ + BRz + SNP—50 mM NaHCO_3_ with 100 μM SNP and 4 μM brassinazole (BRz, a BR biosynthesis inhibitor).
- NaHCO_3_ + cPTIO + EBR—50 mM NaHCO_3_ with 0.2 μM EBR and 150 μM cPTIO (2,4-carboxyphenyl-4,4,5,5-tetramethylimidazoline-1-oxyl-3-oxide, a NO scavenger).
- NaHCO_3_ + L-NAME + EBR—50 mM NaHCO_3_ with 0.2 μM EBR and 200 μM L-NAME (N-nitro-L-arginine methyl ester, an inhibitor of NO synthase).
- NaHCO_3_ + Tungstate + EBR—50 mM NaHCO_3_ with 0.2 μM EBR and 200 μM tungstate (an inhibitor of nitrate reductase).

Each treatment included 20 plants and was organized following a randomized complete block scheme. The nutrient solution was renewed daily and aerated for 20 min each day. qRT-PCR analysis was conducted 16 h post-treatment to assess gene expression, whereas physiological indices were determined after 8 days of exposure.

### 4.2. Determination of Biomass and Root Activity

Shoots and roots were first separated and rinsed thoroughly with deionized water. For biomass measurement, plant samples were initially heated at 105 °C for 20 min so that exogenous enzymes became inactive. Afterwards, they were kept in an oven at 75 °C for about three consecutive days, until the dry mass reached a constant level. The final dry weight was then recorded. Root activity was determined using the triphenyltetrazolium chloride (TTC) reduction method [4]. In brief, 1 g of freshly collected root tissue was placed into 20 mL of 0.5 mM phosphate buffer (pH 7.0) containing 0.4% (*v*/*v*) TTC and incubated at 37 °C for 1 h. To stop the reaction, 2 mL of 1 M H_2_SO_4_ was added. The triphenyl formazan generated (TPF) was extracted with ethyl acetate, and finally, its absorbance was measured at 485 nm using a UV–Vis spectrophotometer (UV-1800, Shimadzu, Kyoto, Japan) spectrophotometer.

### 4.3. Determination of Photosynthetic Apparatus

For photosynthetic measurements, the second fully expanded leaf from each cucumber seedling was chosen. The assessment was carried out using a portable photosynthesis system (LI-6400, LI-COR, Lincoln, NE, USA), which was employed under a photon flux density of 800 μmol m^−2^ s^−1^ together with an ambient CO_2_ concentration of about 340 μmol mol^−1^. Photosynthetic pigments were analyzed after extraction with 95% ethanol. Absorbance of the pigment solutions was then recorded at 663.3, 646.8, and 470 nm with a spectrophotometer, and the data were subsequently used to calculate the concentrations of chlorophyll a, chlorophyll b, and carotenoids individually [62].

### 4.4. Determination of Lipid Peroxidation and Reactive Oxygen Species (ROS) Levels

Malondialdehyde (MDA) was quantified as an indicator of lipid peroxidation following the method of Schmedes and Hølmer [63] with modifications. Briefly, 1 g of fresh leaf tissue was homogenized in 10 mL of 10% (*w*/*v*) trichloroacetic acid (TCA) and centrifuged at 4000× *g* for 10 min. The supernatant (2 mL) was mixed with an equal volume of 0.6% thiobarbituric acid (TBA) solution, incubated in boiling water for 15 min, rapidly cooled, and centrifuged again. The absorbance of the supernatant was recorded at 532, 600, and 450 nm, and MDA concentration was determined using a standard calibration curve.

The generation rate of superoxide anion (O_2_^−^) was measured following the protocol of Elstner and Heupel [64]. Approximately 1 g of fresh tissue was ground in 3 mL phosphate buffer (pH 7.8) and centrifuged at 4000× *g* for 15 min. A 0.5 mL aliquot of the supernatant was combined with 1 mL hydroxylamine hydrochloride and incubated at 25 °C for 1 h. Subsequently, 1 mL of the reaction solution containing 17 mM p-aminobenzene sulfonic acid and 7 mM α-naphthylamine was added and further incubated for 20 min at 25 °C. Absorbance was measured at 530 nm, and the O_2_^−^ content was calculated using a calibration curve prepared with NaNO_2_.

Hydrogen peroxide (H_2_O_2_) content was determined as described by Patterson et al. [65]. Fresh tissue extracts were reacted with the titanium reagent, and the absorbance of the reaction mixture was read at 415 nm. The H_2_O_2_ concentration was calculated by comparison with a standard curve prepared from known concentrations of H_2_O_2_.

### 4.5. Histochemical Staining of MDA and O_2_^−^

MDA staining: Lipid peroxidation was assessed using Schiff’s reagent as outlined by Jambunathan [66]. In brief, 0.05 g of basic fuchsin was first dissolved in a solution consisting of 0.5 mL concentrated HCl mixed with 50 mL distilled water. After that, 0.5 g sodium sulfite was added while stirring continuously until the red coloration disappeared completely. Leaf tissues were immersed in the freshly prepared reagent and incubated for 1 h. Subsequently, they were treated with 80% ethanol at 90 °C until a red or purple precipitate became visible, which indicated the formation of thiobarbituric acid reactive substances (TBARS) linked to MDA buildup.

O_2_^−^ staining: The accumulation of superoxide was examined following the NBT staining approach described by Fryer et al. [67]. Fresh leaves were rinsed thoroughly with distilled water, then transferred into 10 mL centrifuge tubes containing 0.5 mg mL^−1^ NBT prepared in 10 mM phosphate buffer (pH 7.8). To ensure sufficient infiltration, samples were vacuum-treated and later kept in darkness at room temperature for 1 h, thereby enabling O_2_^−^-dependent NBT reduction and the subsequent appearance of blue formazan.

After staining, both MDA and O_2_^−^-treated leaves were heated in 90% ethanol at 90 °C until chlorophyll was fully removed. The decolorized leaves were then photographed under identical conditions to evaluate the staining patterns.

### 4.6. Determination of Antioxidant Enzyme Activities

Fresh cucumber leaves (0.3 g) were homogenized in 3 mL of chilled 50 mM phosphate buffer (pH 7.8) supplemented with 2 mM ascorbic acid, 0.2 mM EDTA, and 2% polyvinylpyrrolidone (PVP). The homogenates were centrifuged at 12,000× *g* for 20 min at 4 °C, and the supernatants were collected for enzymatic assays.

The activity of superoxide dismutase (SOD) was determined following Elstner and Heupel [64], based on its ability to inhibit nitroblue tetrazolium (NBT) photoreduction at 560 nm. Peroxidase (POD) activity was assayed as described by Kochba et al. [68], by monitoring the increase in absorbance at 470 nm due to guaiacol oxidation. Dehydroascorbate reductase (DHAR) activity was assayed according to Shi et al. [69] by monitoring the increase in absorbance at 265 nm due to ascorbate formation in a GSH-dependent reaction. Catalase (CAT) activity was evaluated following Patra et al. [70], by recording the decrease in absorbance at 240 nm caused by H_2_O_2_ decomposition. Ascorbate peroxidase (APX) activity was measured according to Nakano and Asada [71], based on the decline in absorbance at 290 nm due to ascorbic acid oxidation. Glutathione reductase (GR) activity was assayed according to Foyer and Halliwell [72], by monitoring the decrease in absorbance at 340 nm linked to NADPH oxidation during the reduction of GSSG.

### 4.7. Determination of Ascorbate and Glutathione

Ascorbic acid (AsA) and dehydroascorbic acid (DHA) contents were assayed following the method of Hodges et al. [73] with slight adjustments. Fresh cucumber leaves (0.3 g) were homogenized in 2 mL of extraction buffer containing 5% (*v*/*v*) metaphosphoric acid and centrifuged at 12,000× *g* for 20 min at 4 °C. An aliquot of 100 μL supernatant was mixed with 500 μL phosphate buffer (pH 7.4) containing 150 mM potassium iodide (KI) and 5 mM EDTA. For chromogenic development, sequential additions of ethanol (70%) with FeCl_3_ solution, FeCl_3_ (30 g L^−1^), and 10% TCA with 400 μL o-dipyridyl were performed, followed by incubation at 40 °C for 1 h. The absorbance was recorded at 525 nm. DHA concentration was calculated by subtracting the reduced AsA fraction from the total ascorbate pool.

Reduced glutathione (GSH) and oxidized glutathione (GSSG) were determined following the method of Griffith [74] and Ellman [75] using 5,5′-dithiobis-(2-nitrobenzoic acid) (DTNB). Fresh leaf samples (0.3 g) were ground in 3 mL of extraction solution containing 0.5 mM EDTA and 3% TCA, and centrifuged at 15,000× *g* for 10 min at 4 °C. From the resulting supernatant, 0.2 mL was combined with 1.5 mL phosphate buffer (50 mM, pH 7.0) and 0.2 mM DTNB, followed by incubation at 30 °C for 2 min. Absorbance was measured at 412 nm, and concentrations of GSSG and total glutathione were calculated from a calibration curve. GSH levels were obtained by subtracting GSSG values from the total glutathione content.

The contents of AsA, DHA, GSH, and GSSG were initially determined on a fresh weight (FW) basis and then converted to dry weight (DW) according to the corresponding fresh-to-dry weight ratio of each sample.

### 4.8. Quantitative RT–PCR Analysis

Total RNA was extracted from cucumber seedlings using the TRIzol reagent (Invitrogen, Carlsbad, CA, USA) in accordance with the instructions supplied by the manufacturer. The synthesis of first-strand cDNA was carried out with the TransScript All-in-One First-Strand cDNA Synthesis SuperMix for qPCR (TransGen Biotech, Beijing, China), which ensured efficient and complete reverse transcription.

Quantitative real-time PCR (qRT-PCR) was subsequently performed with gene-specific primers (sequences are provided in Table 2). Each reaction was prepared with equal amounts of cDNA template, and all assays were run in triplicate to guarantee reproducibility. PCR reactions were conducted using Power SYBR Green PCR Master Mix (TransGen Biotech, Beijing, China) on an ABI Prism 7900HT Real-Time PCR System (Applied Biosystems, Foster City, CA, USA).

The amplification profile included an initial denaturation, followed by cycles of annealing and extension under optimized conditions for each primer pair. The final concentration of primers was adjusted to 200 nM. Relative expression levels of target genes were calculated using the 2^−ΔΔCt^ method, with Actin employed as the reference gene to normalize variations among samples.

### 4.9. Data Analysis

All data were analyzed using one-way ANOVA (SPSS 22.0, IBM Corp., Armonk, NY, USA), figures were plotted using GraphPad Prism version 9.5.1 (GraphPad Software, San Diego, CA, USA) and significant differences among means were determined by Duncan’s multiple range test at *p* < 0.05.

## Figures and Tables

**Table 1:** Effects of exogenous EBR and NO on photosynthetic parameters of cucumber seedlings under NaHCO_3_ stress.

Treatments	P_n_ (μmol CO_2_ m^−2^ s^−1^)	Chl a (mg g^−1^ DW)	Chl b (mg g^−1^ DW)	Chl a+b (mg g^−1^ DW)	Car (mg g^−1^ DW)
Control	21.43 ± 1.22 ^a^	20.8 ± 1.57 ^a^	6.65 ± 0.47 ^a^	27.45 ± 0.39 ^a^	4.26 ± 0.33 ^a^
NaHCO_3_	11.77 ± 1.32 ^c^	10.76 ± 1.19 ^c^	2.78 ± 0.18 ^c^	13.54 ± 0.19 ^d^	2.6 ± 0.19 ^c^
NaHCO_3_ + SNP	16.73 ± 1.46 ^b^	16.05 ± 1.71 ^b^	4.56 ± 0.34 ^b^	20.62 ± 0.27 ^b^	3.39 ± 0.22 ^b^
NaHCO_3_ + EBR	17.17 ± 1.39 ^b^	16.5 ± 1.44 ^b^	4.69 ± 0.36 ^b^	21.19 ± 0.2 ^b^	3.65 ± 0.31 ^b^
NaHCO_3_ + BRz + SNP	16.13 ± 1.35 ^b^	16.08 ± 0.76 ^b^	4.53 ± 0.37 ^b^	20.63 ± 0.23 ^b^	3.28 ± 0.2 ^b^
NaHCO_3_ + c-PTIO + EBR	13.13 ± 1.25 ^c^	11.16 ± 1.15 ^c^	3.03 ± 0.22 ^c^	14.19 ± 0.15 ^c^	2.67 ± 0.18 ^c^
NaHCO_3_ + L-NAME + EBR	14.33 ± 1.25 ^bc^	11.78 ± 1.53 ^c^	3.07 ± 0.29 ^c^	14.85 ± 0.12 ^c^	2.73 ± 0.21 ^c^
NaHCO_3_ + Tungstate + EBR	13.63 ± 1.40 ^c^	11.32 ± 0.90 ^c^	2.95 ± 0.32 ^c^	14.27 ± 0.17 ^c^	2.71 ± 0.21 ^c^

Note: Pn, net photosynthetic rate; Chl a, chlorophyll a; Chl b, chlorophyll b; Chl a+b, total chlorophyll; Car, carotenoids. Data represent mean ± SD of three replicates. Values in the same column followed by different lowercase letters differ significantly at *p* < 0.05.

**Table 2:** Primers sequences.

Gene Name	Primer Sequences
*Actin*	F:CCCCGATGGGCAGGTAATA R:AAGAGCAGGACGAACAGCAGA
*SOS1*	F: ATCCAACGGAGTGGTAAA R: AACAACGGAATCTGTAATC
*NHX2*	F: AGGGTGTAGTGAATGACG R: GAGAATGCCACTCAAATC
*PIP1:2*	F: CATTATTTACAACCACGACGAAGCA R: GGATTGAAGAAGCATCATGGATTTAGA
*PIP2:4*	F: GCTGCTCTGCTCTCATCTTGCC R: GAAAAATACATGAATAACAGGAGCCCC

## Data Availability

The original contributions presented in this study are included in the article. Further inquiries can be directed to the corresponding author.
